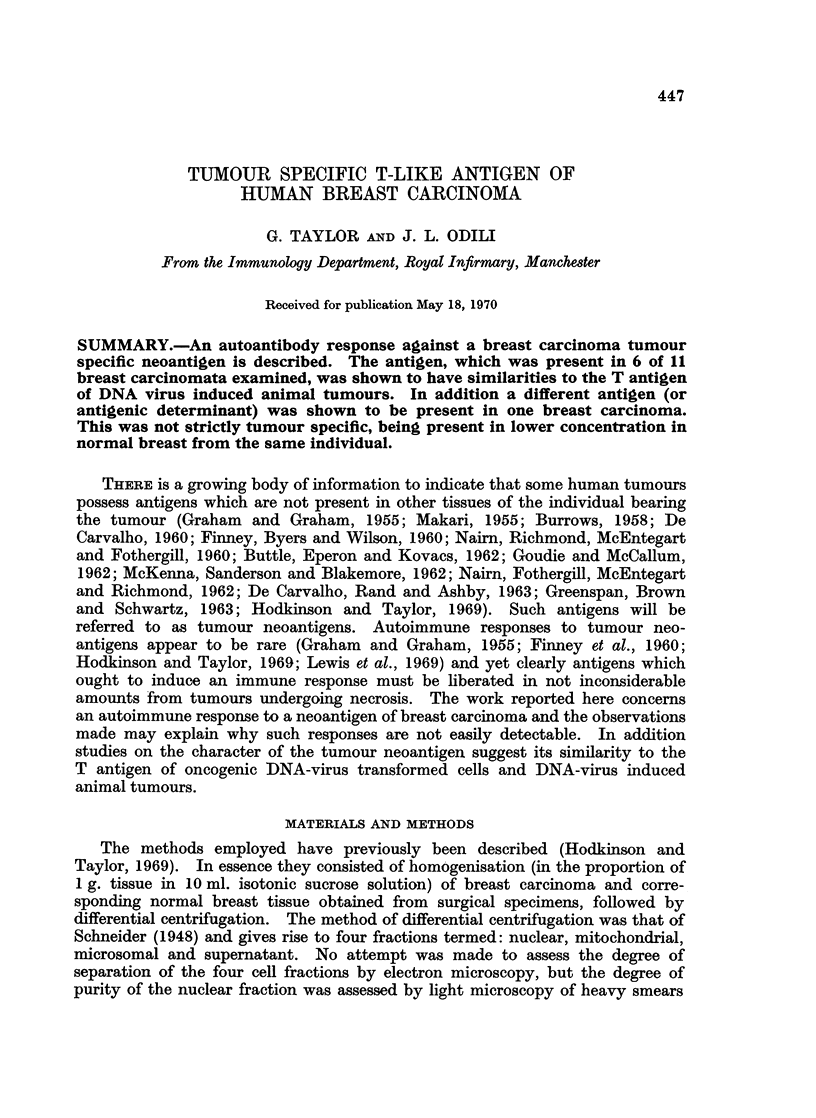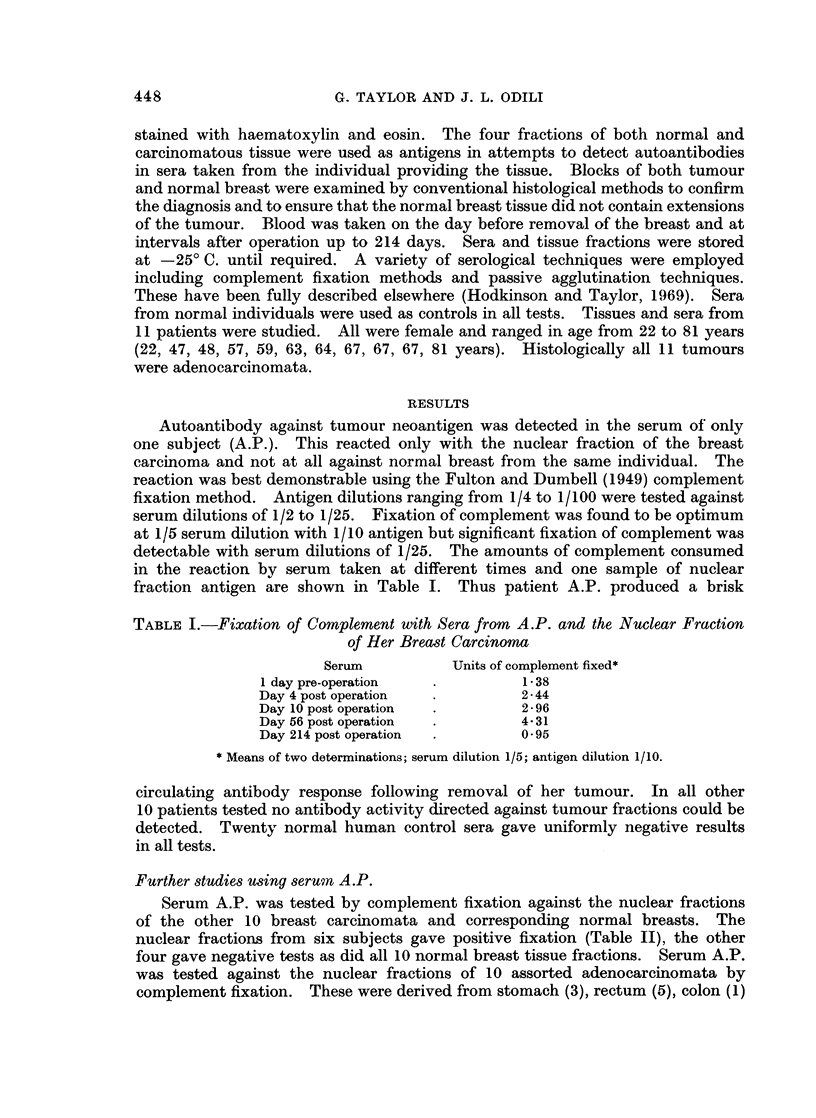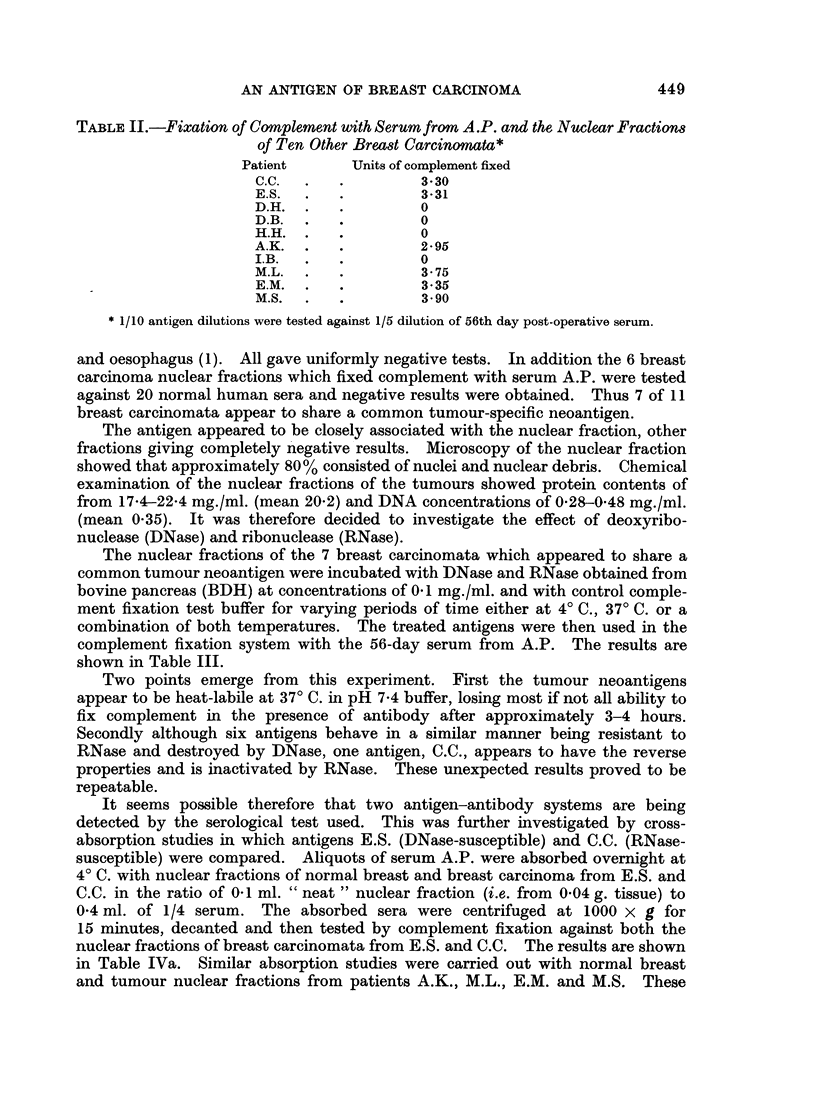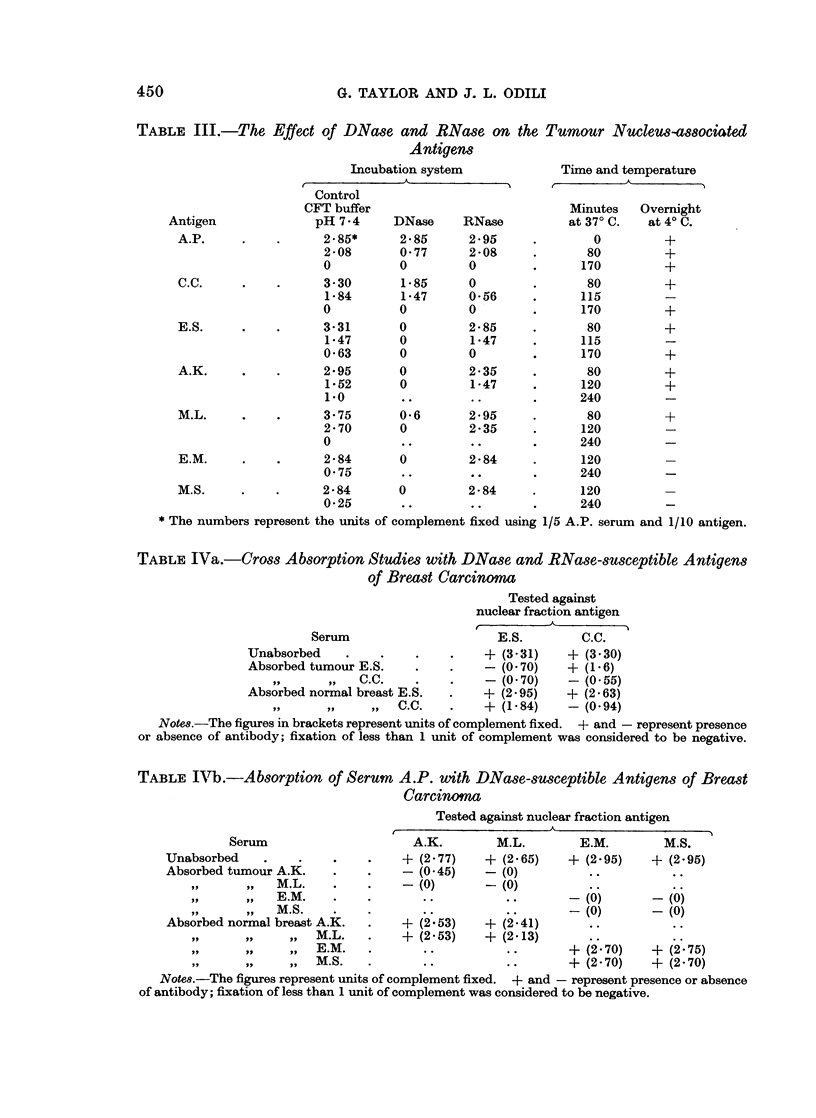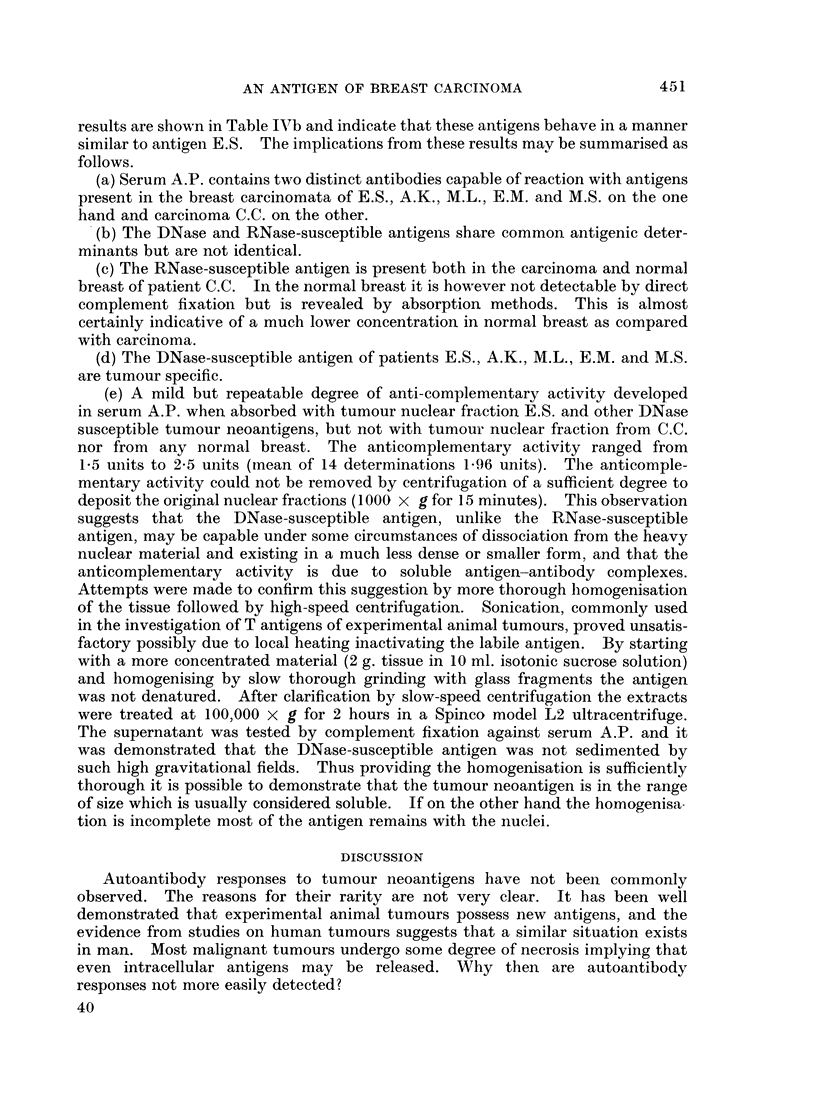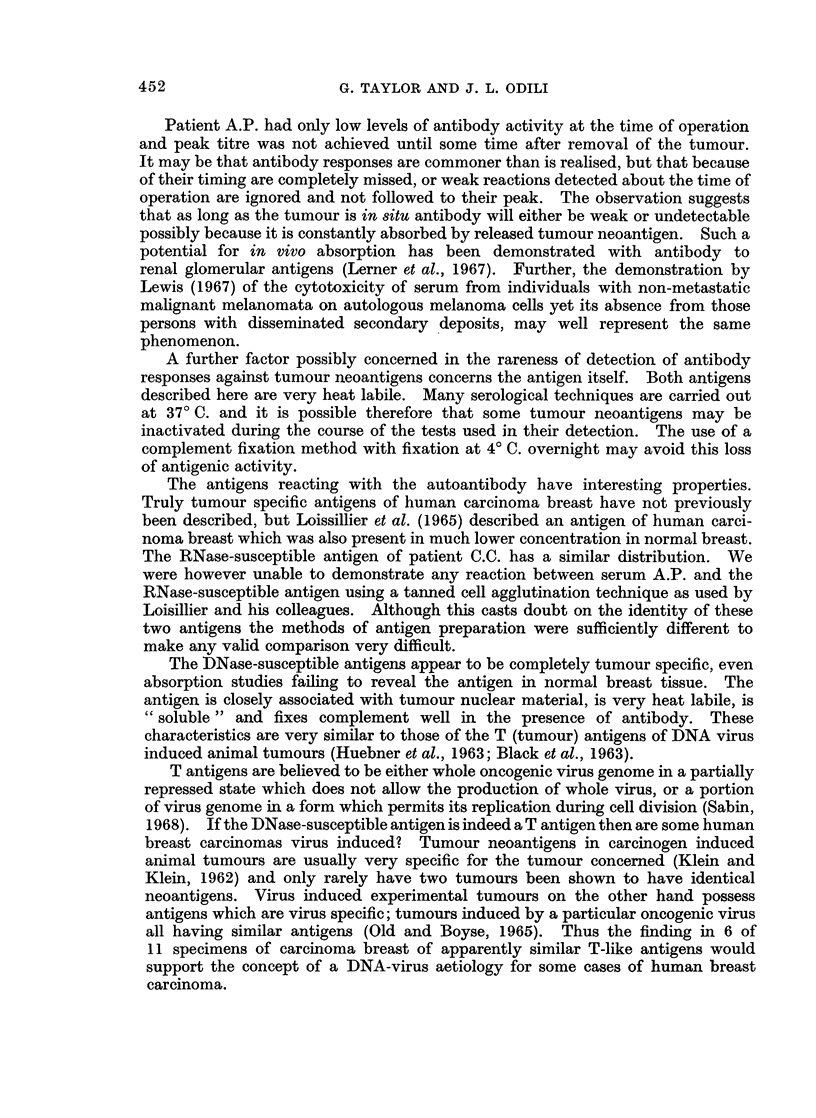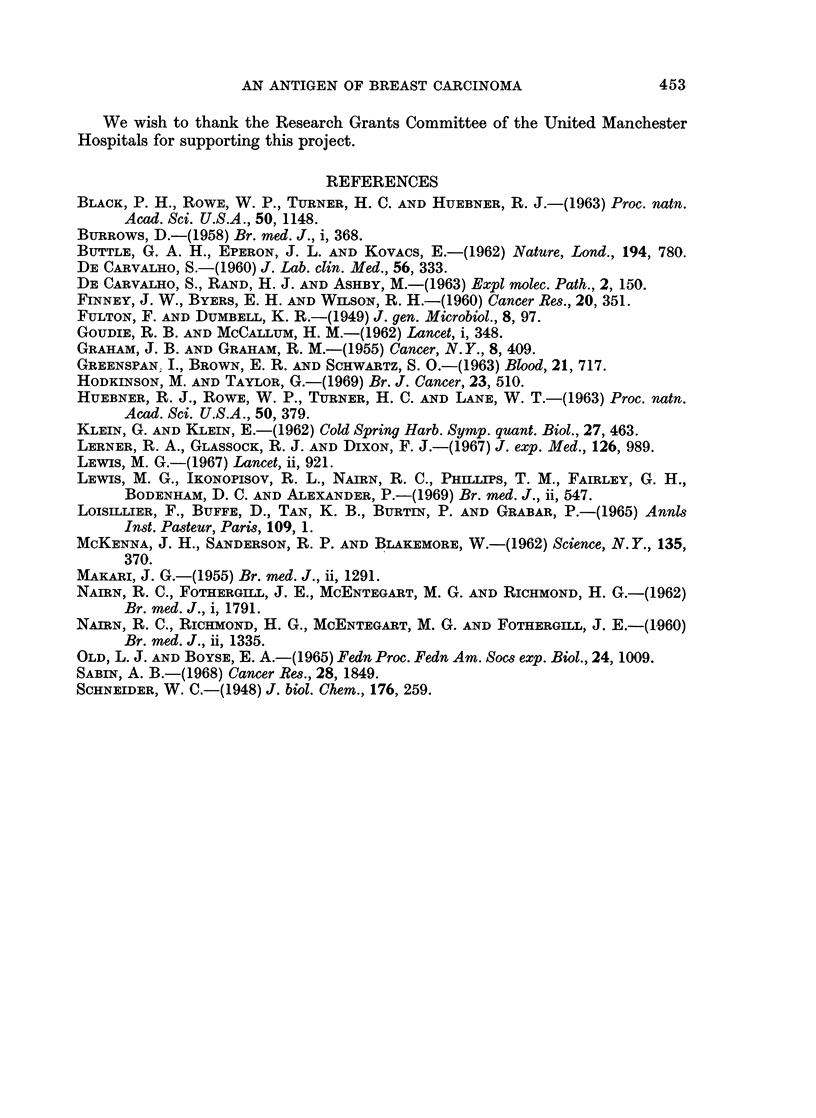# Tumour Specific T-Like Antigen of Human Breast Carcinoma

**DOI:** 10.1038/bjc.1970.53

**Published:** 1970-09

**Authors:** G. Taylor, J. L. Odili

## Abstract

An autoantibody response against a breast carcinoma tumour specific neoantigen is described. The antigen, which was present in 6 of 11 breast carcinomata examined, was shown to have similarities to the T antigen of DNA virus induced animal tumours. In addition a different antigen (or antigenic determinant) was shown to be present in one breast carcinoma. This was not strictly tumour specific, being present in lower concentration in normal breast from the same individual.


### TUMOUR SPECIFIC T-LIKE ANTIGEN OF HUMAN BREAST CARCINOMA

G. TAYLOR AND J. L. ODILI

*From the Immunology Department, Royal Infirmary, Manchester*

Received for publication May 18, 1970

**SUMMARY.—An autoantibody response against a breast carcinoma tumour specific neoantigen is described. The antigen, which was present in 6 of 11 breast carcinomata examined, was shown to have similarities to the T antigen of DNA virus induced animal tumours. In addition a different antigen (or antigenic determinant) was shown to be present in one breast carcinoma. This was not strictly tumour specific, being present in lower concentration in normal breast from the same individual.**

THERE is a growing body of information to indicate that some human tumours possess antigens which are not present in other tissues of the individual bearing the tumour (Graham and Graham, 1955; Makari, 1955; Burrows, 1958; De Carvalho, 1960; Finney, Byers and Wilson, 1960; Nairn, Richmond, McEntegart and Fothergill, 1960; Buttle, Eperon and Kovacs, 1962; Goudie and McCallum, 1962; McKenna, Sanderson and Blakemore, 1962; Nairn, Fothergill, McEntegart and Richmond, 1962; De Carvalho, Rand and Ashby, 1963; Greenspan, Brown and Schwartz, 1963; Hodkinson and Taylor, 1969). Such antigens will be referred to as tumour neoantigens. Autoimmune responses to tumour neoantigens appear to be rare (Graham and Graham, 1955; Finney *et al.*, 1960; Hodkinson and Taylor, 1969; Lewis *et al.*, 1969) and yet clearly antigens which ought to induce an immune response must be liberated in not inconsiderable amounts from tumours undergoing necrosis. The work reported here concerns an autoimmune response to a neoantigen of breast carcinoma and the observations made may explain why such responses are not easily detectable. In addition studies on the character of the tumour neoantigen suggest its similarity to the T antigen of oncogenic DNA-virus transformed cells and DNA-virus induced animal tumours.

### MATERIALS AND METHODS

The methods employed have previously been described (Hodkinson and Taylor, 1969). In essence they consisted of homogenisation (in the proportion of 1 g. tissue in 10 ml. isotonic sucrose solution) of breast carcinoma and corresponding normal breast tissue obtained from surgical specimens, followed by differential centrifugation. The method of differential centrifugation was that of Schneider (1948) and gives rise to four fractions termed: nuclear, mitochondrial, microsomal and supernatant. No attempt was made to assess the degree of separation of the four cell fractions by electron microscopy, but the degree of purity of the nuclear fraction was assessed by light microscopy of heavy smears stained with haematoxylin and eosin. The four fractions of both normal and carcinomatous tissue were used as antigens in attempts to detect autoantibodies in sera taken from the individual providing the tissue. Blocks of both tumour and normal breast were examined by conventional histological methods to confirm the diagnosis and to ensure that the normal breast tissue did not contain extensions of the tumour. Blood was taken on the day before removal of the breast and at intervals after operation up to 214 days. Sera and tissue fractions were stored at —25° C. until required. A variety of serological techniques were employed including complement fixation methods and passive agglutination techniques. These have been fully described elsewhere (Hodkinson and Taylor, 1969). Sera from normal individuals were used as controls in all tests. Tissues and sera from 11 patients were studied. All were female and ranged in age from 22 to 81 years (22, 47, 48, 57, 59, 63, 64, 67, 67, 67, 81 years). Histologically all 11 tumours were adenocarcinomata.

### RESULTS

Autoantibody against tumour neoantigen was detected in the serum of only one subject (A.P.). This reacted only with the nuclear fraction of the breast carcinoma and not at all against normal breast from the same individual. The reaction was best demonstrable using the Fulton and Dumbell (1949) complement fixation method. Antigen dilutions ranging from 1/4 to 1/100 were tested against serum dilutions of 1/2 to 1/25. Fixation of complement was found to be optimum at 1/5 serum dilution with 1/10 antigen but significant fixation of complement was detectable with serum dilutions of 1/25. The amounts of complement consumed in the reaction by serum taken at different times and one sample of nuclear fraction antigen are shown in Table I. Thus patient A.P. produced a brisk

TABLE I.—*Fixation of Complement with Sera from A.P. and the Nuclear Fraction of Her Breast Carcinoma*

| Serum | Units of complement fixed* |
|---|---|
| 1 day pre-operation | 1·38 |
| Day 4 post operation | 2·44 |
| Day 10 post operation | 2·96 |
| Day 56 post operation | 4·31 |
| Day 214 post operation | 0·95 |

* Means of two determinations; serum dilution 1/5; antigen dilution 1/10.

circulating antibody response following removal of her tumour. In all other 10 patients tested no antibody activity directed against tumour fractions could be detected. Twenty normal human control sera gave uniformly negative results in all tests.

### *Further studies using serum A.P.*

Serum A.P. was tested by complement fixation against the nuclear fractions of the other 10 breast carcinomata and corresponding normal breasts. The nuclear fractions from six subjects gave positive fixation (Table II), the other four gave negative tests as did all 10 normal breast tissue fractions. Serum A.P. was tested against the nuclear fractions of 10 assorted adenocarcinomata by complement fixation. These were derived from stomach (3), rectum (5), colon (1)

TABLE II.—*Fixation of Complement with Serum from A.P. and the Nuclear Fractions of Ten Other Breast Carcinomata**

| Patient | Units of complement fixed |
|---|---|
| C.C. . . | 3·30 |
| E.S. . . | 3·31 |
| D.H. . . | 0 |
| D.B. . . | 0 |
| H.H. . . | 0 |
| A.K. . . | 2·95 |
| I.B. . . | 0 |
| M.L. . . | 3·75 |
| E.M. . . | 3·35 |
| M.S. . . | 3·90 |

* 1/10 antigen dilutions were tested against 1/5 dilution of 56th day post-operative serum.

and oesophagus (1). All gave uniformly negative tests. In addition the 6 breast carcinoma nuclear fractions which fixed complement with serum A.P. were tested against 20 normal human sera and negative results were obtained. Thus 7 of 11 breast carcinomata appear to share a common tumour-specific neoantigen.

The antigen appeared to be closely associated with the nuclear fraction, other fractions giving completely negative results. Microscopy of the nuclear fraction showed that approximately 80% consisted of nuclei and nuclear debris. Chemical examination of the nuclear fractions of the tumours showed protein contents of from 17·4–22·4 mg./ml. (mean 20·2) and DNA concentrations of 0·28–0·48 mg./ml. (mean 0·35). It was therefore decided to investigate the effect of deoxyribonuclease (DNase) and ribonuclease (RNase).

The nuclear fractions of the 7 breast carcinomata which appeared to share a common tumour neoantigen were incubated with DNase and RNase obtained from bovine pancreas (BDH) at concentrations of 0·1 mg./ml. and with control complement fixation test buffer for varying periods of time either at 4° C., 37° C. or a combination of both temperatures. The treated antigens were then used in the complement fixation system with the 56-day serum from A.P. The results are shown in Table III.

Two points emerge from this experiment. First the tumour neoantigens appear to be heat-labile at 37° C. in pH 7·4 buffer, losing most if not all ability to fix complement in the presence of antibody after approximately 3–4 hours. Secondly although six antigens behave in a similar manner being resistant to RNase and destroyed by DNase, one antigen, C.C., appears to have the reverse properties and is inactivated by RNase. These unexpected results proved to be repeatable.

It seems possible therefore that two antigen–antibody systems are being detected by the serological test used. This was further investigated by cross-absorption studies in which antigens E.S. (DNase-susceptible) and C.C. (RNase-susceptible) were compared. Aliquots of serum A.P. were absorbed overnight at 4° C. with nuclear fractions of normal breast and breast carcinoma from E.S. and C.C. in the ratio of 0·1 ml. " neat " nuclear fraction (*i.e.* from 0·04 g. tissue) to 0·4 ml. of 1/4 serum. The absorbed sera were centrifuged at 1000 × *g* for 15 minutes, decanted and then tested by complement fixation against both the nuclear fractions of breast carcinomata from E.S. and C.C. The results are shown in Table IVa. Similar absorption studies were carried out with normal breast and tumour nuclear fractions from patients A.K., M.L., E.M. and M.S. These

TABLE III.—*The Effect of DNase and RNase on the Tumour Nucleus-associated Antigens*

| | Incubation system | | | Time and temperature | |
|---|---|---|---|---|---|
| Antigen | Control CFT buffer pH 7·4 | DNase | RNase | Minutes at 37° C. | Overnight at 4° C. |
| A.P. | 2·85* | 2·85 | 2·95 | 0 | + |
| | 2·08 | 0·77 | 2·08 | 80 | + |
| | 0 | 0 | 0 | 170 | + |
| C.C. | 3·30 | 1·85 | 0 | 80 | + |
| | 1·84 | 1·47 | 0·56 | 115 | — |
| | 0 | 0 | 0 | 170 | + |
| E.S. | 3·31 | 0 | 2·85 | 80 | + |
| | 1·47 | 0 | 1·47 | 115 | — |
| | 0·63 | 0 | 0 | 170 | + |
| A.K. | 2·95 | 0 | 2·35 | 80 | + |
| | 1·52 | 0 | 1·47 | 120 | + |
| | 1·0 | .. | .. | 240 | — |
| M.L. | 3·75 | 0·6 | 2·95 | 80 | + |
| | 2·70 | 0 | 2·35 | 120 | — |
| | 0 | .. | .. | 240 | — |
| E.M. | 2·84 | 0 | 2·84 | 120 | — |
| | 0·75 | .. | .. | 240 | — |
| M.S. | 2·84 | 0 | 2·84 | 120 | — |
| | 0·25 | .. | .. | 240 | — |

* The numbers represent the units of complement fixed using 1/5 A.P. serum and 1/10 antigen.

TABLE IVa.—*Cross Absorption Studies with DNase and RNase-susceptible Antigens of Breast Carcinoma*

| | Tested against nuclear fraction antigen | |
|---|---|---|
| Serum | E.S. | C.C. |
| Unabsorbed | + (3·31) | + (3·30) |
| Absorbed tumour E.S. | — (0·70) | + (1·6) |
| ,, ,, C.C. | — (0·70) | — (0·55) |
| Absorbed normal breast E.S. | + (2·95) | + (2·63) |
| ,, ,, ,, C.C. | + (1·84) | — (0·94) |

*Notes.*—The figures in brackets represent units of complement fixed. + and — represent presence or absence of antibody; fixation of less than 1 unit of complement was considered to be negative.

TABLE IVb.—*Absorption of Serum A.P. with DNase-susceptible Antigens of Breast Carcinoma*

| | Tested against nuclear fraction antigen | | | |
|---|---|---|---|---|
| Serum | A.K. | M.L. | E.M. | M.S. |
| Unabsorbed | + (2·77) | + (2·65) | + (2·95) | + (2·95) |
| Absorbed tumour A.K. | — (0·45) | — (0) | .. | .. |
| ,, ,, M.L. | — (0) | — (0) | .. | .. |
| ,, ,, E.M. | .. | .. | — (0) | — (0) |
| ,, ,, M.S. | .. | .. | — (0) | — (0) |
| Absorbed normal breast A.K. | + (2·53) | + (2·41) | .. | .. |
| ,, ,, ,, M.L. | + (2·53) | + (2·13) | .. | .. |
| ,, ,, ,, E.M. | .. | .. | + (2·70) | + (2·75) |
| ,, ,, ,, M.S. | .. | .. | + (2·70) | + (2·70) |

*Notes.*—The figures represent units of complement fixed. + and — represent presence or absence of antibody; fixation of less than 1 unit of complement was considered to be negative.

results are shown in Table IVb and indicate that these antigens behave in a manner similar to antigen E.S. The implications from these results may be summarised as follows.

(a) Serum A.P. contains two distinct antibodies capable of reaction with antigens present in the breast carcinomata of E.S., A.K., M.L., E.M. and M.S. on the one hand and carcinoma C.C. on the other.

(b) The DNase and RNase-susceptible antigens share common antigenic determinants but are not identical.

(c) The RNase-susceptible antigen is present both in the carcinoma and normal breast of patient C.C. In the normal breast it is however not detectable by direct complement fixation but is revealed by absorption methods. This is almost certainly indicative of a much lower concentration in normal breast as compared with carcinoma.

(d) The DNase-susceptible antigen of patients E.S., A.K., M.L., E.M. and M.S. are tumour specific.

(e) A mild but repeatable degree of anti-complementary activity developed in serum A.P. when absorbed with tumour nuclear fraction E.S. and other DNase susceptible tumour neoantigens, but not with tumour nuclear fraction from C.C. nor from any normal breast. The anticomplementary activity ranged from 1·5 units to 2·5 units (mean of 14 determinations 1·96 units). The anticomplementary activity could not be removed by centrifugation of a sufficient degree to deposit the original nuclear fractions (1000 $\times$ $g$ for 15 minutes). This observation suggests that the DNase-susceptible antigen, unlike the RNase-susceptible antigen, may be capable under some circumstances of dissociation from the heavy nuclear material and existing in a much less dense or smaller form, and that the anticomplementary activity is due to soluble antigen–antibody complexes. Attempts were made to confirm this suggestion by more thorough homogenisation of the tissue followed by high-speed centrifugation. Sonication, commonly used in the investigation of T antigens of experimental animal tumours, proved unsatisfactory possibly due to local heating inactivating the labile antigen. By starting with a more concentrated material (2 g. tissue in 10 ml. isotonic sucrose solution) and homogenising by slow thorough grinding with glass fragments the antigen was not denatured. After clarification by slow-speed centrifugation the extracts were treated at 100,000 $\times$ $g$ for 2 hours in a Spinco model L2 ultracentrifuge. The supernatant was tested by complement fixation against serum A.P. and it was demonstrated that the DNase-susceptible antigen was not sedimented by such high gravitational fields. Thus providing the homogenisation is sufficiently thorough it is possible to demonstrate that the tumour neoantigen is in the range of size which is usually considered soluble. If on the other hand the homogenisation is incomplete most of the antigen remains with the nuclei.

### DISCUSSION

Autoantibody responses to tumour neoantigens have not been commonly observed. The reasons for their rarity are not very clear. It has been well demonstrated that experimental animal tumours possess new antigens, and the evidence from studies on human tumours suggests that a similar situation exists in man. Most malignant tumours undergo some degree of necrosis implying that even intracellular antigens may be released. Why then are autoantibody responses not more easily detected?

Patient A.P. had only low levels of antibody activity at the time of operation and peak titre was not achieved until some time after removal of the tumour. It may be that antibody responses are commoner than is realised, but that because of their timing are completely missed, or weak reactions detected about the time of operation are ignored and not followed to their peak. The observation suggests that as long as the tumour is *in situ* antibody will either be weak or undetectable possibly because it is constantly absorbed by released tumour neoantigen. Such a potential for *in vivo* absorption has been demonstrated with antibody to renal glomerular antigens (Lerner *et al.*, 1967). Further, the demonstration by Lewis (1967) of the cytotoxicity of serum from individuals with non-metastatic malignant melanomata on autologous melanoma cells yet its absence from those persons with disseminated secondary deposits, may well represent the same phenomenon.

A further factor possibly concerned in the rareness of detection of antibody responses against tumour neoantigens concerns the antigen itself. Both antigens described here are very heat labile. Many serological techniques are carried out at 37° C. and it is possible therefore that some tumour neoantigens may be inactivated during the course of the tests used in their detection. The use of a complement fixation method with fixation at 4° C. overnight may avoid this loss of antigenic activity.

The antigens reacting with the autoantibody have interesting properties. Truly tumour specific antigens of human carcinoma breast have not previously been described, but Loissillier *et al.* (1965) described an antigen of human carcinoma breast which was also present in much lower concentration in normal breast. The RNase-susceptible antigen of patient C.C. has a similar distribution. We were however unable to demonstrate any reaction between serum A.P. and the RNase-susceptible antigen using a tanned cell agglutination technique as used by Loisillier and his colleagues. Although this casts doubt on the identity of these two antigens the methods of antigen preparation were sufficiently different to make any valid comparison very difficult.

The DNase-susceptible antigens appear to be completely tumour specific, even absorption studies failing to reveal the antigen in normal breast tissue. The antigen is closely associated with tumour nuclear material, is very heat labile, is "soluble" and fixes complement well in the presence of antibody. These characteristics are very similar to those of the T (tumour) antigens of DNA virus induced animal tumours (Huebner *et al.*, 1963; Black *et al.*, 1963).

T antigens are believed to be either whole oncogenic virus genome in a partially repressed state which does not allow the production of whole virus, or a portion of virus genome in a form which permits its replication during cell division (Sabin, 1968). If the DNase-susceptible antigen is indeed a T antigen then are some human breast carcinomas virus induced? Tumour neoantigens in carcinogen induced animal tumours are usually very specific for the tumour concerned (Klein and Klein, 1962) and only rarely have two tumours been shown to have identical neoantigens. Virus induced experimental tumours on the other hand possess antigens which are virus specific; tumours induced by a particular oncogenic virus all having similar antigens (Old and Boyse, 1965). Thus the finding in 6 of 11 specimens of carcinoma breast of apparently similar T-like antigens would support the concept of a DNA-virus aetiology for some cases of human breast carcinoma.

We wish to thank the Research Grants Committee of the United Manchester Hospitals for supporting this project.

### REFERENCES

BLACK, P. H., ROWE, W. P., TURNER, H. C. AND HUEBNER, R. J.—(1963) *Proc. natn. Acad. Sci. U.S.A.*, **50**, 1148.
BURROWS, D.—(1958) *Br. med. J.*, i, 368.
BUTTLE, G. A. H., EPERON, J. L. AND KOVACS, E.—(1962) *Nature, Lond.*, **194**, 780.
DE CARVALHO, S.—(1960) *J. Lab. clin. Med.*, **56**, 333.
DE CARVALHO, S., RAND, H. J. AND ASHBY, M.—(1963) *Expl molec. Path.*, **2**, 150.
FINNEY, J. W., BYERS, E. H. AND WILSON, R. H.—(1960) *Cancer Res.*, **20**, 351.
FULTON, F. AND DUMBELL, K. R.—(1949) *J. gen. Microbiol.*, **8**, 97.
GOUDIE, R. B. AND MCCALLUM, H. M.—(1962) *Lancet*, i, 348.
GRAHAM, J. B. AND GRAHAM, R. M.—(1955) *Cancer, N.Y.*, **8**, 409.
GREENSPAN, I., BROWN, E. R. AND SCHWARTZ, S. O.—(1963) *Blood*, **21**, 717.
HODKINSON, M. AND TAYLOR, G.—(1969) *Br. J. Cancer*, **23**, 510.
HUEBNER, R. J., ROWE, W. P., TURNER, H. C. AND LANE, W. T.—(1963) *Proc. natn. Acad. Sci. U.S.A.*, **50**, 379.
KLEIN, G. AND KLEIN, E.—(1962) *Cold Spring Harb. Symp. quant. Biol.*, **27**, 463.
LERNER, R. A., GLASSOCK, R. J. AND DIXON, F. J.—(1967) *J. exp. Med.*, **126**, 989.
LEWIS, M. G.—(1967) *Lancet*, ii, 921.
LEWIS, M. G., IKONOPISOV, R. L., NAIRN, R. C., PHILLIPS, T. M., FAIRLEY, G. H., BODENHAM, D. C. AND ALEXANDER, P.—(1969) *Br. med. J.*, ii, 547.
LOISILLIER, F., BUFFE, D., TAN, K. B., BURTIN, P. AND GRABAR, P.—(1965) *Annls Inst. Pasteur, Paris*, **109**, 1.
MCKENNA, J. H., SANDERSON, R. P. AND BLAKEMORE, W.—(1962) *Science, N.Y.*, **135**, 370.
MAKARI, J. G.—(1955) *Br. med. J.*, ii, 1291.
NAIRN, R. C., FOTHERGILL, J. E., MCENTEGART, M. G. AND RICHMOND, H. G.—(1962) *Br. med. J.*, i, 1791.
NAIRN, R. C., RICHMOND, H. G., MCENTEGART, M. G. AND FOTHERGILL, J. E.—(1960) *Br. med. J.*, ii, 1335.
OLD, L. J. AND BOYSE, E. A.—(1965) *Fedn Proc. Fedn Am. Socs exp. Biol.*, **24**, 1009.
SABIN, A. B.—(1968) *Cancer Res.*, **28**, 1849.
SCHNEIDER, W. C.—(1948) *J. biol. Chem.*, **176**, 259.